# Effects of COVID-19 on Blood Culture Contamination at a Tertiary Care Academic Medical Center

**DOI:** 10.1128/spectrum.00277-22

**Published:** 2022-03-30

**Authors:** Brianna Sacchetti, Justin Travis, Lisa L. Steed, Ginny Webb

**Affiliations:** a Division of Natural Sciences and Engineering, University of South Carolina Upstate, Spartanburg, South Carolina, USA; b Department of Psychology, University of South Carolina Upstate, Spartanburg, South Carolina, USA; c Department of Pathology and Laboratory Medicine, Medical University of South Carolina, Charleston, South Carolina, USA; University of Cincinnati

**Keywords:** blood culture, contamination, phlebotomy, nursing, COVID-19

## Abstract

The COVID-19 pandemic has changed health care, from increased needs of personal protective equipment (PPE) to overloaded staff and influxes of patients. Blood cultures are frequently used to detect bloodstream infections in critically ill patients, but it is unknown whether the COVID-19 pandemic has had an impact on blood culture contamination rates. A total of 88,332 blood cultures taken over a 33-month period were analyzed to compare blood culture contamination rates before the COVID-19 pandemic to rates during the pandemic. A significant increase in the average number of monthly nurse-drawn and peripherally collected cultures occurred after the start of the pandemic, but there was a decrease in the average number of phlebotomy cultures. A significant increase in contamination rates (*P* < 0.001) was found in all nonemergency hospital departments during the COVID-19 pandemic, increasing from 2.1% to 2.5%. Increased rates during the COVID-19 pandemic were also found for nursing staff (2.0% to 2.4%) and both peripheral (2.1% to 2.5%) and indwelling line draws (1.1% to 1.7). The number of cultures drawn monthly increased in acute adult departments and both adult and pediatric emergency departments. Blood culture contamination rates in adult acute, adult emergency, and pediatric intensive care units increased after the start of the pandemic by 23%, 75%, and 59%, respectively. A positive correlation was found between blood culture contamination rates and COVID-19 incidence rates. Additional periodic staff training on proper blood collection technique and awareness of the workload of health care workers are recommended to decrease contamination rates during the COVID-19 pandemic.

**IMPORTANCE** Understanding factors that contribute to blood culture contamination is important in order to take steps to limit contamination events. Here, we examine the effect the COVID-19 pandemic has had on blood culture contamination rates and specifically detail the effects based on the staff, draw types, and unit types. The conclusions provided here can be used as hospitals and laboratories navigate the COVID-19 pandemic or other times of high patient volume.

## INTRODUCTION

A blood culture is a diagnostic test frequently used in hospitalized patients to detect bloodstream infections. Rapid identification of bloodstream infections is imperative in the hospital setting because of the high risk of progression t osepsis, a life-threatening condition in which the body attempts to fight a bloodstream infection, causing severe inflammation throughout the body and, in some cases, causing organ failure and death (1). A 2001 study by Angus et al. estimated an annual total of 751,000 cases of sepsis with about a 28.6% mortality rate in the United States alone (2). When skin and environmental bacteria are mistakenly introduced into the sample, the culture becomes contaminated. A contaminated blood culture is defined as a single positive blood culture containing skin and environmental organisms associated with inadequate skin antisepsis (such as *Bacillus* spp., *Corynebacterium* spp., *Cutibacterium* spp., *Micrococcus* sp., and coagulase-negative staphylococci) (3).

This contamination leads to an inaccurate diagnosis resulting in unnecessary antibiotic treatment and additional laboratory testing to identify the false positive. This prolongs the patient's length of stay and increases hospital costs. Coagulase-negative staphylococci are responsible for about 85% of contamination events, with most other contaminants being natural skin microflora (4, 5). Blood culture contamination (BCC) is extremely costly to health care institutions; a study by Dargére et al. projected that costs associated with blood culture contamination accumulated to about $2,000,000 annually per facility (6).

In December 2019, the virus SARS-CoV-2, which causes coronavirus disease 19 (COVID-19), was discovered and has infected over 412 million and has claimed the lives of over 5.8 million worldwide as of 15 February 2022 (7). The first case of COVID-19 in the United States was identified in January 2020, with the first case in South Carolina identified in March 2020 (8). The severity of COVID-19 symptoms is highly variable among patients, but the most common symptoms include fever, cough, shortness of breath, and, in more extreme cases, heart failure, acute respiratory distress syndrome, and multiple organ failure (9). There is overlap of some symptoms of COVID-19 and bloodstream infections, complicating clinical presentation differentiation between the two. Accurate blood culture results are vitally important for prompt and correct treatment. The COVID-19 pandemic has had a dramatic negative effect on health care workers due to the influx of critically ill patients that are infected with the virus. The increase in patient load poses a constant threat to hospital systems worldwide as critical care unit capacities become overloaded with concomitant shortages of personal protective equipment, ventilators, medications, and other crucial medical supplies (10). A recent study by Yu et al. surveyed patients that tested positive for COVID-19 and found that the presence of bloodstream infections in COVID-19 patients is low, but blood culture contamination is more likely (11). Other, more specialized studies have shown that the likelihood of central line-associated bloodstream infections is significantly higher in patients positive for COVID-19 than in patients negative for COVID-19 (12, 13). Blood cultures are performed frequently on patients with severe COVID-19 infection because once a cytokine storm or irregular immune response is detected, bloodstream infection must immediately be ruled out before treatment begins (14, 15). If increased cases of COVID-19 contributed to rises in blood culture contamination rates, hospitals can expect skyrocketing costs and needed staff. In this study, we aim to determine if the COVID-19 pandemic has had a significant impact on BCC rates by examining contamination rate data from a tertiary care academic medical center over a 33-month period immediately before and after the start of the COVID-19 pandemic. If a notable change is found, extra attention should be given to the proper collection of blood cultures during the COVID-19 pandemic.

## RESULTS

**FIG 1:**
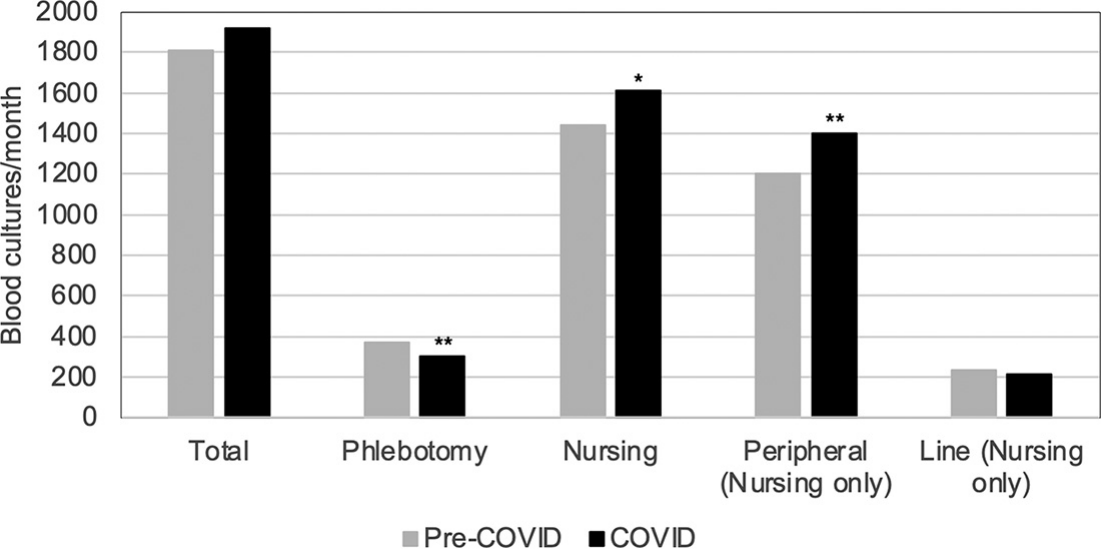
Average number of blood cultures collected per month before COVID and during COVID. Pre-COVID cultures were collected between July 2018 and February 2020. Cultures obtained during COVID were from March 2020 to March 2021. Cultures collected in emergency departments are not included. *P* values were calculated using *t* test. *, *P* < 0.01; **, *P* < 0.001.

The COVID-19 pandemic did not appear to have an effect on the average number of cultures collected per month for the adult and pediatric hospitals combined (excluding emergency), as no statistical significance was found when comparing the pre-COVID period to the COVID period. However, when comparing the pre- and post-COVID average draws based on what staff collected them, nurses collected significantly more cultures after COVID-19 had begun than before the pandemic (*P* < 0.01), while phlebotomy staff collected significantly fewer cultures after the start of the pandemic (*P* < 0.001) (Fig. 1). When nurse-drawn cultures were separated into line and peripheral draws, there was a significant increase in number of peripheral cultures after the onset of COVID-19 (*P* < 0.001), while no change was apparent in line draws (Fig. 1).

The inpatient contamination rates of the main hospitals (excluding emergency departments [EDs]) were significantly increased after the onset of the COVID-19 pandemic (2.5%) compared to pre-COVID-19 (2.1%) [*X*^2^(1) = 12.805; *P* < 0.001] (Table 1). Nurses also showed increased contamination rates after the start of COVID-19 (2.4% versus 2.0%) [*X*^2^(1) = 12.789; *P* < 0.001], while the rates for phlebotomy staff showed no change (Table 1). Both line and peripheral draws performed by nursing staff had increased contamination rates after the start of the pandemic, as line draw contamination increased from 1.1% to 1.7% [*X*^2^(1) = 5.183; *P* < 0.05], and peripheral draw contamination increased from 2.1% to 2.5% [*X*^2^(1) = 7.791; *P* < 0.01] (Table 1).

**TABLE 1:** Blood culture contamination rates before and during COVID^*a*^

Type of culture	Pre-COVID		COVID		*P* value
	No. of noncontaminated cultures	No. (%) of contaminated cultures	No. of noncontaminated cultures	No. (%) of contaminated cultures	
Total	35,410	751 (2.1)	24,271	626 (2.5)	<0.001
Phlebotomy	7,187	186 (2.5)	3,836	116 (2.9)	0.194
Nursing	28,223	565 (2.0)	20,435	510 (2.4)	<0.001
Peripheral (nursing only)	23,552	513 (2.1)	17,700	462 (2.5)	<0.01
Line (nursing only)	4,671	52 (1.1)	2,735	48 (1.7)	<0.05

a All cultures included pre-COVID were collected between July 2018 and February 2020, and all COVID cultures were collected between March 2020 and March 2021. Emergency department cultures were not included in these data.

**FIG 2:**
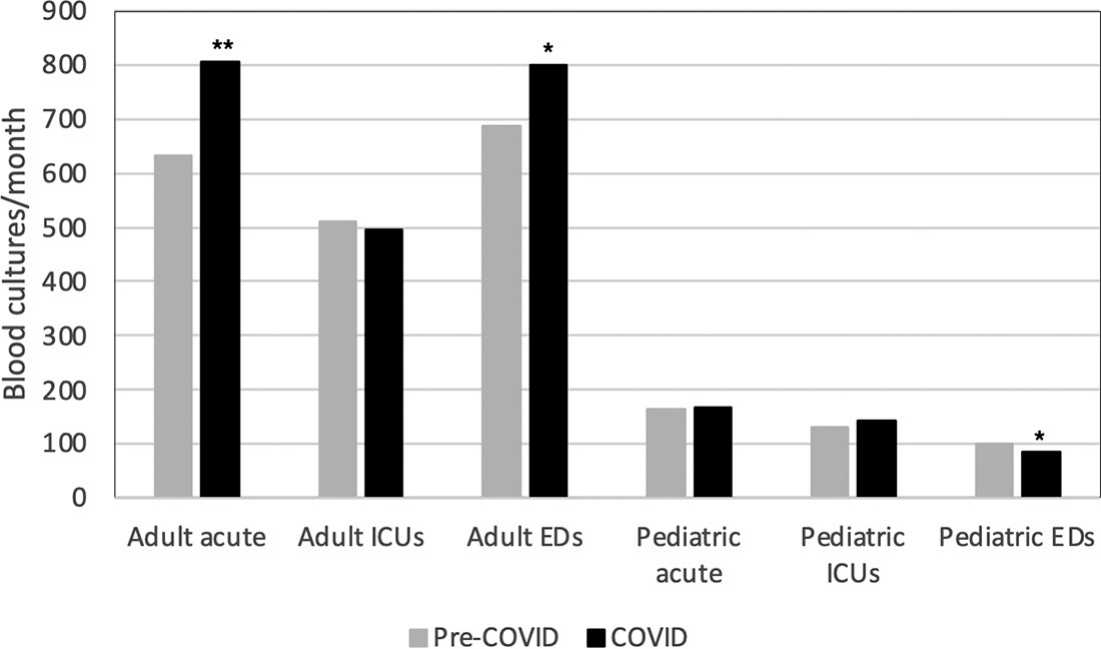
Average number of blood cultures collected monthly from pre-COVID to COVID. Cultures were separated by hospital department and then compared from July 2018 to February 2020 in the pre-COVID period from March 2020 to March 2021 in the COVID period. *, *P* < 0.01; **, *P* < 0.0001.

Various units were compared in the adult and pediatric hospitals, intensive care units (ICUs), EDs, and acute units that include all remaining units. Average monthly blood cultures from pre-COVID-19 and COVID-19 periods showed a significant increase in cultures in the acute adult units, from an average of 632 monthly cultures collected before COVID to a monthly average of 805 cultures (*P* < 0.0001). In addition, the adult ERs saw a significant increase from 687 to 801 average monthly cultures pre- to post-COVID onset (*P* < 0.01) (Fig. 2). The adult ICUs did not have a change in average monthly cultures after the start of COVID-19 (Fig. 2). The only significant change from before and after the pandemic in the pediatric departments was a decrease in average monthly cultures collected in the pediatric ED, from 101 monthly cultures pre-COVID-19 to 85 monthly cultures during COVID-19 (*P* < 0.01) (Fig. 2).

After the onset of COVID-19, contamination rates in the pediatric ICUs significantly increased from 2.2% to 3.5% [*X*^2^(1) = 7.622; *P* < 0.01], but no difference was found in the adult ICUs (Table 2). A notable increase was also observed in the adult EDs from pre-COVID (2.0%) to after the onset of COVID (3.5%) [*X*^2^(1) = 56.201; *P* < 0.00001], but no significance was found in the pediatric ED (Table 2). In the adult acute units, a significant increase in contamination rates from 2.1% pre-COVID to 2.6% after the start of COVID [*X*^2^(1) = 5.766; *P* < 0.05] occurred, but no remarkable changes were found in the pediatric acute units (Table 2).

**TABLE 2:** Blood culture contamination rates in separate departments pre- and during COVID^*a*^

Department	Pre-COVID		COVID		*P* value
	No. of noncontaminated cultures	No. (%) of contaminated cultures	No. of noncontaminated cultures	No. (%) of contaminated cultures	
Adult acute	12,365	269 (2.1)	10,188	273 (2.6)	<0.05
Adult ICUs	10,027	194 (1.9)	6,283	144 (2.2)	0.127
Adult EDs	13,468	270 (2.0)	10,039	367 (3.5)	<0.00001
Pediatric acute	3,257	45 (1.4)	2,159	27 (1.2)	0.68
Pediatric ICUs	2,574	57 (2.2)	1,805	66 (3.5)	<0.01
Pediatric EDs	1,971	43 (2.1)	1,080	26 (2.4)	0.695

a Cultures from all hospital departments were compared from the period of July 2018 to February 2020 before the pandemic to the period of March 2020 to March 2021 during the pandemic.

**FIG 3:**
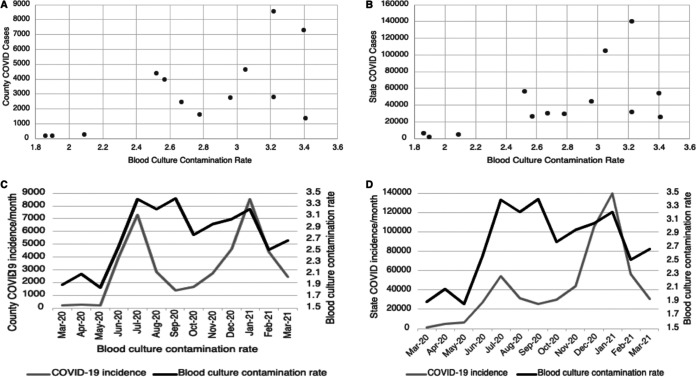
Relationship between COVID-19 incidence rate and blood culture contamination rate. (A) Correlation between county-wide COVID-19 incidence rate and blood culture contamination rates. (*R* = 0.640; *P* < 0.05; 95% confidence interval [CI,] 0.14 to 0.88). (B) Correlation between statewide COVID-19 incidence rate and blood culture contamination rates. (*R* = 0.575; *P* < 0.05; 95% CI, 0.035 to 0.86). (C and D) Overlay of COVID-19 county (C) and state (D) incidence rates with BCC rates.

Community COVID-19 infection rates for the county in which this hospital is located were also compared to BCC rates. A positive correlation was found between the monthly number of positive COVID-19 cases and monthly hospital BCC rates (*r *= 0.640; *P* < 0.05) (Fig. 3A). In addition, a positive correlation was present between South Carolina’s monthly positive COVID-19 cases and the monthly hospital blood culture contamination rates (*r *= 0.575; *P* < 0.05) (Fig. 3B). Further supporting this relationship, Fig. 3C and D show overlays of county-wide and statewide COVID-19 incidence and BCC rates to demonstrate their patterns over time.

## DISCUSSION

The COVID-19 pandemic has led to many drastic changes in the medical field, including the need for additional personal protective equipment (PPE) use and precautions that are necessary in order to keep patients and hospital staff safe, not to mention the influx of patients infected with COVID-19, overwhelming hospitals worldwide. With these extensive safeguards, it may be expected to see a decrease in BCC rates because more effort is used to stop the spread of bacteria and viruses; however, exhausted staff may be more prone to technical errors during blood culture collection, allowing more opportunity for contamination. Our results have indicated that the COVID-19 pandemic has had a negative impact on BCC in areas throughout this tertiary care academic medical center. COVID-19 has been present in the United States for over almost 2 years and shows no signs of disappearing in the near future, so it is necessary to acknowledge the issue of spikes in contamination and changes that must be made to maintain low contamination rates.

In this study, the average number of cultures drawn per month was compared in the pre-COVID-19 period to the COVID-19 period. When analyzing cultures, we can assume that increases in the number of cultures imply increases in hospitalized patient volumes, even though multiple cultures may be drawn on some patients over time. The number of cultures drawn in all hospital departments (excluding the EDs) did not significantly increase after the start of the pandemic. However, a significant increase was found in the average number of cultures collected by nursing staff. This increase in blood culture collection is expected due to the increases in hospitalized patients seen around the world from the COVID-19 pandemic (16). A similar study by Sepulveda et al. found a 34.8% increase in the number of blood cultures ordered from pre-COVID to after the start of the COVID surge, as well as a significantly lower prevalence of bloodstream infections in COVID-positive patients than in COVID-negative patients (17). On the contrary, a significant decrease in the monthly average draws was found in phlebotomy staff. Hospitals have continuously struggled with staff shortages since the beginning of the COVID pandemic, as clinical staff are at constant risk of contracting the virus (18). This decrease in monthly draws collected by phlebotomy staff seen at this facility may be a result of a medical personnel shortage or staff changes due to only essential personnel working during early stages of the pandemic, but further research would be necessary to make recommendations for additional phlebotomy staffing. When distinguishing the types of nurse draws, there was a significant increase in the monthly average peripheral draws, which was expected because the majority of blood cultures are drawn from a peripheral site rather than indwelling lines. No significant increase was present in the number of line draws during the COVID-19 pandemic, likely an advantage to this institution, as other hospitals have seen concerning increases in central line-associated infections, with one study of 78 hospitals reporting an increase of 51% since before the pandemic (12).

The COVID pandemic has hit hospital staff especially hard, with shortages of PPE, numerous critically ill patients, almost constant exposure to COVID infection themselves, and high levels of stress; heightened levels of contamination are not unexpected (19). All inpatient units at this medical center saw a significant increase in BCC from 2.1% pre-COVID to 2.5% during COVID. A similar study based on Detroit hospitals found a 29% increase in BCC compared to the prepandemic period (20). After comparing BCC rates from before and after the start of the pandemic, only nursing staff were found to have a significant increase in contamination, from 2.0% prepandemic to 2.4% post-pandemic onset. After finding a 19% increase in contamination post-COVID onset, a study by LeRose et al. surveyed their nursing staff and found that the leading causes of contamination were thought to be inadequate skin preparation, not changing the site from which serial cultures are drawn, and neglecting to draw multiple cultures (13). Additionally, our data show a significant increase was found in peripheral draw contamination, from 2.1% to 2.5%, as well as indwelling line draw contamination rates rising from 1.1% to 1.7%. A focus on educating staff on correct aseptic technique and skin preparation when collecting blood cultures would likely be an effective intervention to reduce contamination during the pandemic, as other facilities have seen progress (21). From the pre-COVID period to the COVID period, phlebotomy staff did not show any significant increase in contamination rates since the COVID-19 period, possibly a result of medical staff shortages that have been seen around the world since the start of the pandemic (22). It is important to consider that the nursing staff in this study collected over four times as many blood cultures as phlebotomy staff, and other studies have shown that overwhelming workloads and increased hospital volumes have a negative impact on BCC rates (23, 24). The COVID-19 pandemic has had a dramatic negative effect on health care workers due to the influx of critically ill patients that are infected with the virus. For a few months early during the pandemic, nonessential employees were furloughed, elective procedures were postponed, and hospital admissions were restricted to COVID-positive patients, emergent surgical and trauma, and other critically ill patients whose care could not be delayed. Overall workload dropped dramatically. However, as the pandemic continued, the medical center moved to various degrees of modified operations, which affected the number and types of patients admitted. Unfortunately, many health care workers left the facility, and new staff were hired, requiring training and creating additional stress. A distinguishing feature of this study is the finding that the type of staff collecting a culture plays a significant role in COVID-19-related BCC changes. Ensuring phlebotomy staff availability or that other staff are adequately trained in BC collection is important for minimizing contamination events.

Different types of departments in the adult and pediatric hospitals, including acute, ICUs, and EDs, were then analyzed to determine if there were changes in the monthly average draws collected from prepandemic to the pandemic period. In the adult hospital, a significant increase in average monthly draws was found in both the acute units and EDs. Despite record hospitalizations during the pandemic, many EDs have seen significant decreases in patient volumes, as fewer patients are coming in to be seen for conditions that are not COVID-related or that may appear to be non-life-threatening (25–28). Results from this medical center’s pediatric ED support this claim, as they had a significant decrease from 101 to just 85 monthly draws on average after the start of the pandemic. Other pediatric ICUs have seen significant cases of children with severe COVID infection; a study by Shekerdemian et al. found that 38% of children positive for COVID-19 in ICUs required a ventilator for treatment, although life-threatening infection in children was found to be significantly less frequent than that in adults (29). Since patients with severe COVID-19 are more likely to develop sepsis (30), it was unexpected to find no significant increase in monthly blood draws in either the adult or pediatric ICUs. In addition, no changes were found in monthly draws in the pediatric acute departments, which is supported by similar studies that conclude that children’s hospital admissions decreased in 2020 after the start of the pandemic compared to previous years (31). Pelletier et al. suggests that the decrease in pediatric hospital admissions could be a result of inadequate focus on pediatric care during COVID, especially at the start of the pandemic, when virtually nothing was known about the effects that COVID-19 infection has on children, so further research on the topic may be beneficial to keeping pediatric hospital admissions steady (31).

BCC rates were then compared from the pre-COVID period to the COVID period. Similar to the average number of monthly cultures, the contamination rates increased after the start of COVID in both the adult acute units and adult EDs. Contamination rates in the acute units increased from 2.1% pre-COVID to 2.6% during COVID, while rates in the EDs skyrocketed from 2.0% to 3.5%, resulting in 23% and 75% increases, respectively. It is important to note that phlebotomists in this hospital do not cover the EDs. Given our findings that phlebotomist-collected cultures did not have increased BCC rates during the pandemic while nurse-collected cultures did, the type of staff collecting the BC may contribute to the finding of increased rates in the ED. In addition, the increase in the number of blood cultures may also contribute to this increased rate in the ED. Orders for blood culture draws that are not medically necessary are especially high in fast-paced emergency departments or units with high patient volumes, when physicians need to obtain a lot of information in a limited amount of time (23, 32). One emergency department that aimed to eliminate draws for unneeded blood cultures through staff education on process-of-care measures before submitting orders for a blood culture saved their facility an estimated $765,000 annually by reducing their overall draws performed by over 20% (32). These results highlight the importance of avoiding ordering unnecessary blood cultures even when presented with an overwhelming volume of patients throughout the ongoing COVID-19 pandemic. A rise in contamination rates was also seen in the pediatric ICUs, from 2.2% prepandemic to 3.5% after the onset, a 59% increase. The unpredictability of COVID-19 infections could also be responsible for the rise in contamination rates because other studies, such as one by Thelen et al., found that the prevalence of true bacteremia in COVID-positive patients is significantly lower than that of patients with other more common illnesses, such as the flu (33). If bloodstream infections are less likely in COVID patients, unless bloodstream infection is suspected, collecting blood cultures from COVID patients should be considered with caution, as unnecessary laboratory testing provides opportunity to contaminants. No increases in contamination rates were found in the adult ICUs, a possible result of extensive PPE use and disinfection precautions that have been implemented in ICUs worldwide since the beginning of the pandemic (34). The same results were seen in the pediatric acute units and pediatric ED, both departments that may pose risk of exposure to COVID-19 and have taken extra precautions since the start of COVID-19.

This study also sought to determine if a relationship was present between BCC rates and community COVID-19 cases. When contamination rates in this facility were compared to positive COVID cases in the same county as the hospital, a positive correlation was found. Heightened contamination rates among a variety of other clinical errors can be expected during a pandemic, as hospital staff experience psychological stress with escalating COVID cases, especially within such close proximity to the hospital (35). A positive correlation was also found between BCC rates for this facility and positive COVID cases in the state. Our data also shows the trend of COVID-19 incidence and BCC rates over time is similar, suggesting that BCC rates increased in times of COVID-19 surges. This finding suggests BCC rate changes may either be due to increased hospitalizations or due to changes in unit arrangements and staffing. It is recommended to emphasize the importance of high levels of stress and overwhelming workloads of health care workers during the ongoing pandemic, which can have an adverse impact on their performance (36).

It is important to note that while this study provides important comparisons to determine the effect of the COVID-19 pandemic on BCC rates, the overall contamination rates in this hospital are low and mostly fall well below the recommended 3% rate. Many studies have concluded that heavy workloads have a negative impact of staff performance and patient care, and SARS-CoV-2 has tested the capacities of many hospitals that are simply not prepared for the increase in critically ill patients (37–39). It is also imperative that hospital staff are provided the proper education and supplies to complete their job effectively. Additional staff training on proper blood culture collection, especially during the pandemic, can increase staff confidence and lead to more effective safety enforcement (40).

This study was presented with multiple limitations and setbacks, especially with the COVID-19 pandemic interfering with hospital systems and data collection. One limitation is a blood culture was assigned as contaminated based on organism isolation, and it is possible the organism could be the cause of a true infection and therefore misclassified as BCC. Individual patient data were not available regarding patient COVID-19 status; therefore, we could not compare BCC rates in COVID-19 patients to other patients. For future studies, it would be recommended to collect data on which patients were COVID positive. Future research may be interested in comparing the BCC rates in units treating COVID patients versus units treating non-COVID patients in addition to studying the frequency of true bloodstream infections in COVID-positive patients. Physicians could be educated to avoid ordering blood cultures for every fever spike in COVID patients and order them only when there are other clinical indications of a bacterial infection leading to bacteremia. It may be beneficial to conduct a survey on nursing staff to gain feedback from the frontline workers on what can be changed in hospital systems to improve staff morale during the pandemic as well as recommendations for improving contamination rates.

## MATERIALS AND METHODS

### Data collection.

A total of 88,322 blood cultures were analyzed from a South Carolina tertiary care academic medical center during the pre-COVID-19 period of 1 July 2018 to 28 February 2020 and the COVID-19 period of 1 March 2020 to 31 March 2021. This medical center has 700 beds with 2 adult inpatient towers serving different patient needs (30 total units), a pediatric hospital (16 total units) with its own emergency department (ED), a level 1 trauma center, and a chest pain ED. BD Bactec Plus aerobic, anaerobic, and Peds Plus bottles were used. A blood culture is defined as one blood specimen submitted for culture from one blood draw, regardless of how many bottles the specimen is inoculated into. Contaminated cultures were identified using the CLSI M47 criteria for identifying a contaminated blood culture, a positive blood culture containing skin, and environmental organisms associated with inadequate skin antisepsis (3). Recovery of *Bacillus* spp., *Corynebacterium* spp., *Cutibacterium* spp., *Staphylococci* spp. except S. aureus and S. lugdunensis, and *Micrococcus* spp. from single positive blood cultures were categorized as contaminants. BCC rates were calculated by dividing the number of contaminated cultures by the total number of cultures drawn and multiplying by 100 to obtain a percentage. All positive blood cultures were included.

Data were collected and analyzed by the laboratory and sorted by staff type, unit type, and collection type. Cultures were separated by unit in adult and pediatric hospitals (61,058) and adult and pediatric EDs (27,264). Blood cultures were drawn from patients both positive and negative for COVID-19 that were suspected of having a bloodstream infection. The data provided included cultures collected by nursing and phlebotomy staff, and specifics were given on both peripheral and line draws completed by nurse staff only, as phlebotomists only used peripheral draws. These data were used to compare the changes in monthly blood culture collection from prepandemic to the first year of the pandemic. Statistical analyses were performed to determine if a relationship is present between the COVID-19 pandemic and BCC for different staff types, methods of collection, and unit types in this hospital system. In addition, community COVID-19 rates were compared to hospital contamination rates.

In order to protect both staff and patients from contracting and spreading COVID-19, the medical center followed all precautions implemented by the CDC as they developed throughout the course of this study, which included, but were not limited to, the use of masks, additional PPE, and extensive disinfection of frequently used surfaces.

COVID-19 incidence rates at the county and state level were obtained from the South Carolina Department of Health and Environmental Control’s COVID-19 data dashboard (41).

### Statistical analysis.

Our analyses were performed using IBM’s Statistical Package for the Social Sciences (SPSS). Chi-square tests of independence were performed on aggregated data (e.g., nursing or phlebotomy, adult or pediatric) to compare BCC rates pre-COVID-19 to rates during the COVID-19 period. Additionally, we tested the linear relationship between community infection rates and BCC rates by calculating Pearson’s correlation coefficient and for differences in the monthly number of cultures tested before and during the COVID-19 pandemic by conducting an independent-samples *t* test.
